# [1,1′-Bis(dicyclo­hexyl­phosphino)cobalto­cenium-κ^2^ *P*,*P*′]chlorido(η^5^-cyclo­penta­dien­yl)ruthenium(II) hexa­fluorido­phosphate

**DOI:** 10.1107/S1600536810001790

**Published:** 2010-01-30

**Authors:** Jian-Guo Hou

**Affiliations:** a Department of Biotic Environment, Nanchang Instiute of Technology, Nanchang 330013, People’s Republic of China

## Abstract

In the title structure, [CoRu(C_5_H_5_)(C_17_H_26_P)_2_Cl]PF_6_, the Ru^II^ atom is bonded to a cyclo­penta­dienyl ring, a Cl atom and two P atoms of the chelating 1,1′-bis­(dicyclo­hexyl­phosphino)cobaltocenium (di-cypc) ligand, leading to a three-legged piano-stool coordination. Part of the PF_6_^−^ counter-anion is disordered over two positions, with a site-occupancy ratio of 0.898 (7):0.102 (7). The components are linked by C—H⋯F and C—H⋯Cl hydrogen bonds.

## Related literature

For synthesis and crystal structure of the related compound [Ru(η^5^-C_5_H_5_(dppc)]PF_6_ (dppc = 1,1′-bis­(diphenyl­phos­phino)cobaltocenium), see: Wu *et al.* (2006 ▶).
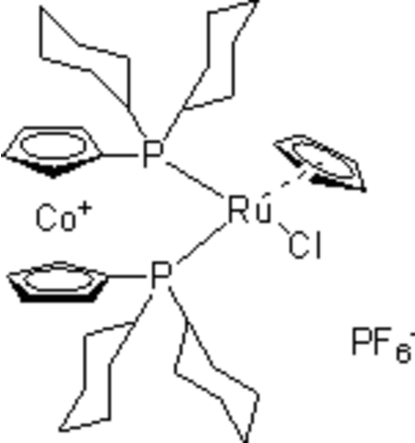


## Experimental

### 

#### Crystal data

[CoRu(C_5_H_5_)(C_17_H_26_P)_2_Cl]PF_6_
*M* *_r_* = 928.21
Monoclinic, 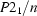

*a* = 14.0095 (16) Å
*b* = 16.9051 (19) Å
*c* = 17.987 (2) Å
β = 110.107 (2)°
*V* = 4000.3 (8) Å^3^
*Z* = 4
Mo *K*α radiation
μ = 1.04 mm^−1^
*T* = 292 K
0.30 × 0.20 × 0.20 mm

#### Data collection

Bruker SMART CCD area-detector diffractometer
Absorption correction: multi-scan (*SADABS*; Sheldrick, 2004 ▶) *T*_min_ = 0.747, *T*_max_ = 0.820
45838 measured reflections
9503 independent reflections
7178 reflections with *I* > 2σ(*I*)
*R*_int_ = 0.139

#### Refinement

*R*[*F* ^2^ > 2σ(*F* ^2^)] = 0.046
*wR*(*F* ^2^) = 0.108
*S* = 0.96
9503 reflections
489 parameters
H-atom parameters constrained
Δρ_max_ = 0.75 e Å^−3^
Δρ_min_ = −0.54 e Å^−3^

### 

Data collection: *SMART* (Bruker, 2001 ▶); cell refinement: *SAINT* (Bruker, 2001 ▶); data reduction: *SAINT*; program(s) used to solve structure: *SHELXS97* (Sheldrick, 2008 ▶); program(s) used to refine structure: *SHELXL97* (Sheldrick, 2008 ▶); molecular graphics: *SHELXTL* (Sheldrick, 2008 ▶); software used to prepare material for publication: *SHELXTL*.

## Supplementary Material

Crystal structure: contains datablocks I, global. DOI: 10.1107/S1600536810001790/wm2296sup1.cif

Structure factors: contains datablocks I. DOI: 10.1107/S1600536810001790/wm2296Isup2.hkl

Additional supplementary materials:  crystallographic information; 3D view; checkCIF report

## Figures and Tables

**Table:** 

Ru1—C35	2.186 (3)
Ru1—C36	2.195 (3)
Ru1—C39	2.199 (3)
Ru1—C38	2.206 (3)
Ru1—C37	2.208 (4)
Ru1—P2	2.3282 (8)
Ru1—P1	2.3297 (7)
Ru1—Cl1	2.4372 (7)

**Table:** 

P2—Ru1—P1	97.67 (3)
P2—Ru1—Cl1	89.17 (3)
P1—Ru1—Cl1	90.70 (3)
C10—P1—C23	98.49 (14)
C23—P1—Ru1	114.06 (11)
C5—P2—C17	98.20 (14)
C5—P2—Ru1	118.51 (9)

**Table 2:** Hydrogen-bond geometry (Å, °)

*D*—H⋯*A*	*D*—H	H⋯*A*	*D*⋯*A*	*D*—H⋯*A*
C9—H9⋯Cl1	0.93	2.65	3.284 (3)	127
C6—H6⋯F1^i^	0.93	2.32	3.176 (4)	152
C3—H3⋯F4^ii^	0.93	2.47	3.181 (6)	134

Symmetry codes: (i) 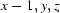; (ii) 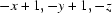.

**Fig. 1.**
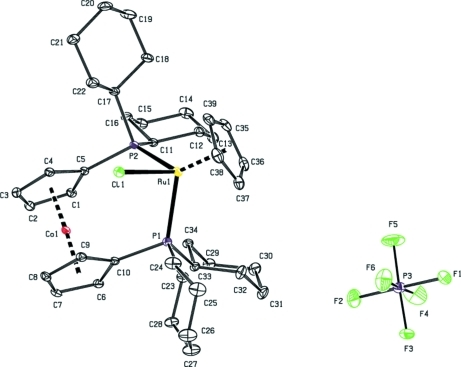
The molecular structure of (I), with atom labels and 30% probability displacement ellipsoids for all non-H atoms. H atoms are omitted for clarity. For the PF6- anion only one orientation is shown.

**Fig. 2.**
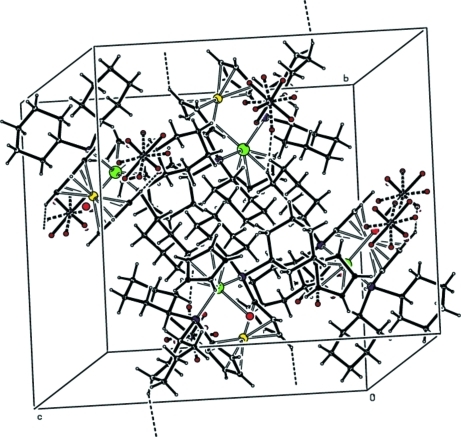
The packing of (I), showing the molecules connected by C—H···F and C—H···Cl hydrogen bonds (dashed lines).

## supplementary crystallographic information

### Comment

The molecular structure of [Ru(η^5^-C_5_H_5_)(di-cypc)Cl][PF_6_] (di-cypc is 1,1'-bis(dicyclohexylphosphino) cobaltocenium), (I), is shown in Fig. 1. The configuration about the Rh(II) atom is similar to that of the related compound [Ru(η^5^-C_5_H_5_(dppc)][PF_6_] (dppc is 1,1'-bis(diphenylphosphino)cobaltocenium (Wu *et al.*, 2006). The Ru—P distances of 2.3297 (7) and 2.3282 (8) Å are somewhat longer than those in the dppc analogue (2.2656 (11) and 2.2695 (11) Å, respectively, which is due to the bulky cyclohexyl ligand. On the other hand, this results in a smaller P—Ru—P chelate angle of 97.67 (3)° in (I) title compound compared with 99.70 (4)° in [Ru(η^5^-C_5_H_5_(dppc)][PF_6_]. The Cp rings are symmetrically disposed about the Co atom and the dihedral ange between the two Cp rings is 3.36 (17)° (4.1 (1)° in [Ru(η^5^-C_5_H_5_(dppc)][PF_6_]).

Molecules are linked by C—H···F and C—H···Cl hydrogen bonds, as shown in Fig. 2.

### Experimental

The synthetic method has been described earlier (Wu *et al.*, 2006). A mixture of [Ru(η^5^-C_5_H_5_)(PPh_3_)_2_Cl] (0.544 g, 0.75 mmol) and [di-cypc][PF_6_] (0.575 g, 0.8 mmol, slight excess) was refluxed in CH_2_Cl_2_ (30 ml) for 24 h. The solution was evaporated, and the residue was chromatographed on alumina by elution with CH_2_Cl_2_ to give the title compound as a green solid (0.619, 63%). Crystals appropriate for data collection were obtained by slow evaporation from dichloromethane and hexane solution at room temperature.

### Refinement

All H atoms were placed in geometrically idealized positions and constrained to ride on their parent atoms with C—H = 0.93 Å or 0.97 Å with *U*_iso_(H) = 1.2*U*_eq_(C). The PF^-^ anion is disordered over two positons. The final occupancies were refined to a ratio of 0.898 (7):0.102 (7) for the major and minor components, respectively, using also the DFIX commant to constrain the P—F distances.

### Crystal data

**Table:**

[CoRu(C_5_H_5_)(C_17_H_26_P)_2_Cl]PF_6_	*F*(000) = 1912
*M**_r_* = 928.21	*D*_x_ = 1.541 Mg m^−^^3^
Monoclinic, *P*2_1_/*n*	Mo *K*α radiation, λ = 0.71073 Å
Hall symbol: -P 2yn	Cell parameters from 6151 reflections
*a* = 14.0095 (16) Å	θ = 2.3–25.7°
*b* = 16.9051 (19) Å	µ = 1.03 mm^−^^1^
*c* = 17.987 (2) Å	*T* = 292 K
β = 110.107 (2)°	Block, black
*V* = 4000.3 (8) Å^3^	0.30 × 0.20 × 0.20 mm
*Z* = 4	

### Data collection

**Table:**

Bruker SMART CCD area-detector diffractometer	9503 independent reflections
Radiation source: fine-focus sealed tube	7178 reflections with *I* > 2σ(*I*)
graphite	*R*_int_ = 0.139
φ and ω scans	θ_max_ = 28.0°, θ_min_ = 1.6°
Absorption correction: multi-scan (*SADABS*; Sheldrick, 2004)	*h* = −18→18
*T*_min_ = 0.747, *T*_max_ = 0.820	*k* = −21→22
45838 measured reflections	*l* = −23→23

### Refinement

**Table:**

Refinement on *F*^2^	Primary atom site location: structure-invariant direct methods
Least-squares matrix: full	Secondary atom site location: difference Fourier map
*R*[*F*^2^ > 2σ(*F*^2^)] = 0.046	Hydrogen site location: inferred from neighbouring sites
*wR*(*F*^2^) = 0.108	H-atom parameters constrained
*S* = 0.96	*w* = 1/[σ^2^(*F*_o_^2^) + (0.0494*P*)^2^] where *P* = (*F*_o_^2^ + 2*F*_c_^2^)/3
9503 reflections	(Δ/σ)_max_ < 0.001
489 parameters	Δρ_max_ = 0.75 e Å^−^^3^
0 restraints	Δρ_min_ = −0.54 e Å^−^^3^

### Special details

**Table:**

Geometry. All e.s.d.'s (except the e.s.d. in the dihedral angle between two l.s. planes) are estimated using the full covariance matrix. The cell e.s.d.'s are taken into account individually in the estimation of e.s.d.'s in distances, angles and torsion angles; correlations between e.s.d.'s in cell parameters are only used when they are defined by crystal symmetry. An approximate (isotropic) treatment of cell e.s.d.'s is used for estimating e.s.d.'s involving l.s. planes.
Refinement. Refinement of *F*^2^ against ALL reflections. The weighted *R*-factor *wR* and goodness of fit *S* are based on *F*^2^, conventional *R*-factors *R* are based on *F*, with *F* set to zero for negative *F*^2^. The threshold expression of *F*^2^ > σ(*F*^2^) is used only for calculating *R*-factors(gt) *etc*. and is not relevant to the choice of reflections for refinement. *R*-factors based on *F*^2^ are statistically about twice as large as those based on *F*, and *R*- factors based on ALL data will be even larger.

### Fractional atomic coordinates and isotropic or equivalent isotropic displacement parameters (Å^2^)

**Table:**

	*x*	*y*	*z*	*U*_iso_*/*U*_eq_	Occ. (<1)
Ru1	0.556184 (17)	0.318279 (13)	0.174161 (14)	0.03634 (8)	
Co1	0.27620 (3)	0.44068 (2)	0.04044 (2)	0.03548 (10)	
Cl1	0.57558 (6)	0.42143 (4)	0.08576 (5)	0.04739 (19)	
P1	0.46046 (5)	0.40312 (4)	0.22280 (4)	0.03324 (16)	
P2	0.42263 (6)	0.26549 (4)	0.06948 (4)	0.03325 (16)	
P3	0.98214 (7)	0.37054 (6)	0.13022 (6)	0.0576 (2)	
F1	1.07889 (19)	0.40569 (15)	0.19711 (15)	0.0894 (7)	
F2	0.8878 (2)	0.3330 (2)	0.06407 (19)	0.1320 (12)	
F3	0.9724 (3)	0.3074 (2)	0.18898 (18)	0.1047 (16)	0.898 (7)
F4	0.9097 (3)	0.4248 (3)	0.1547 (4)	0.170 (2)	0.898 (7)
F5	0.9920 (4)	0.4375 (4)	0.0729 (3)	0.176 (3)	0.898 (7)
F6	1.0572 (3)	0.3183 (3)	0.1041 (3)	0.138 (2)	0.898 (7)
F3'	0.9364 (19)	0.3730 (18)	0.1922 (14)	0.074 (9)*	0.102 (7)
F4'	0.938 (2)	0.4442 (15)	0.1044 (16)	0.068 (8)*	0.102 (7)
F5'	1.024 (2)	0.3797 (19)	0.0653 (17)	0.077 (9)*	0.102 (7)
F6'	1.028 (2)	0.2862 (15)	0.1421 (18)	0.077 (9)*	0.102 (7)
C1	0.2150 (2)	0.33276 (15)	0.00525 (18)	0.0415 (7)	
H1	0.1864	0.2996	0.0331	0.050*	
C2	0.1622 (3)	0.39175 (18)	−0.04980 (19)	0.0512 (8)	
H2	0.0934	0.4038	−0.0643	0.061*	
C3	0.2331 (3)	0.42879 (18)	−0.07845 (18)	0.0531 (8)	
H3	0.2189	0.4692	−0.1158	0.064*	
C4	0.3289 (3)	0.39428 (16)	−0.04090 (17)	0.0434 (7)	
H4	0.3888	0.4092	−0.0485	0.052*	
C5	0.3199 (2)	0.33255 (15)	0.01089 (16)	0.0354 (6)	
C6	0.2576 (2)	0.47172 (16)	0.14378 (18)	0.0412 (7)	
H6	0.2242	0.4429	0.1715	0.049*	
C7	0.2126 (3)	0.52909 (17)	0.08465 (19)	0.0479 (8)	
H7	0.1446	0.5441	0.0667	0.058*	
C8	0.2881 (2)	0.55932 (16)	0.05777 (19)	0.0465 (7)	
H8	0.2788	0.5979	0.0191	0.056*	
C9	0.3811 (2)	0.52080 (15)	0.09986 (18)	0.0407 (7)	
H9	0.4433	0.5299	0.0935	0.049*	
C10	0.3627 (2)	0.46562 (15)	0.15355 (16)	0.0349 (6)	
C11	0.3404 (2)	0.18618 (15)	0.08930 (17)	0.0361 (6)	
H11	0.295	0.2145	0.1114	0.043*	
C12	0.3969 (2)	0.12615 (16)	0.15388 (19)	0.0446 (7)	
H12A	0.4412	0.0939	0.1351	0.054*	
H12B	0.4388	0.1543	0.2007	0.054*	
C13	0.3222 (3)	0.07303 (19)	0.1752 (2)	0.0594 (9)	
H13A	0.3596	0.0334	0.2130	0.071*	
H13B	0.2838	0.1046	0.2001	0.071*	
C14	0.2496 (3)	0.03233 (18)	0.1032 (2)	0.0624 (10)	
H14A	0.2006	0.0020	0.1186	0.075*	
H14B	0.2871	−0.0042	0.0820	0.075*	
C15	0.1937 (3)	0.09141 (19)	0.0395 (2)	0.0589 (9)	
H15A	0.1506	0.1245	0.0587	0.071*	
H15B	0.1506	0.0632	−0.0069	0.071*	
C16	0.2687 (3)	0.14333 (17)	0.01675 (19)	0.0482 (7)	
H16A	0.3080	0.1107	−0.0065	0.058*	
H16B	0.2315	0.1819	−0.0225	0.058*	
C17	0.4554 (3)	0.22163 (18)	−0.0142 (2)	0.0495 (8)	
H17	0.390	0.210	−0.056	0.059*	
C18	0.5113 (3)	0.14241 (19)	0.0066 (2)	0.0643 (10)	
H18A	0.5786	0.1512	0.0451	0.077*	
H18B	0.4746	0.1079	0.0305	0.077*	
C19	0.5209 (4)	0.1021 (2)	−0.0667 (3)	0.0865 (14)	
H19A	0.5583	0.0530	−0.0512	0.104*	
H19B	0.4537	0.0895	−0.1034	0.104*	
C20	0.5743 (4)	0.1540 (2)	−0.1068 (3)	0.0778 (12)	
H20A	0.5738	0.1289	−0.1555	0.093*	
H20B	0.6445	0.1605	−0.0729	0.093*	
C21	0.5242 (4)	0.2340 (2)	−0.1256 (2)	0.0764 (12)	
H21A	0.4573	0.2281	−0.1655	0.092*	
H21B	0.5642	0.2678	−0.1472	0.092*	
C22	0.5138 (3)	0.2734 (2)	−0.0520 (2)	0.0629 (10)	
H22A	0.5809	0.2838	−0.0140	0.075*	
H22B	0.4790	0.3236	−0.0668	0.075*	
C23	0.5363 (2)	0.48242 (18)	0.2890 (2)	0.0483 (8)	
H23	0.573	0.4546	0.338	0.058*	
C24	0.6185 (3)	0.5190 (2)	0.2663 (3)	0.0739 (12)	
H24A	0.5892	0.5541	0.2215	0.089*	
H24B	0.6564	0.4781	0.2506	0.089*	
C25	0.6908 (3)	0.5662 (2)	0.3366 (3)	0.0805 (13)	
H25A	0.7240	0.5304	0.3799	0.097*	
H25B	0.7429	0.5915	0.3207	0.097*	
C26	0.6322 (4)	0.6288 (2)	0.3643 (3)	0.0892 (14)	
H26A	0.6772	0.6522	0.4130	0.107*	
H26B	0.6116	0.6704	0.3248	0.107*	
C27	0.5423 (4)	0.5986 (2)	0.3780 (3)	0.0846 (14)	
H27A	0.5015	0.6431	0.3836	0.101*	
H27B	0.5638	0.5698	0.4277	0.101*	
C28	0.4770 (3)	0.5453 (2)	0.3138 (2)	0.0571 (9)	
H28A	0.4405	0.5771	0.2680	0.068*	
H28B	0.4271	0.5198	0.3322	0.068*	
C29	0.3897 (2)	0.36368 (16)	0.28573 (16)	0.0372 (6)	
H29	0.346	0.4066	0.2925	0.045*	
C30	0.4575 (3)	0.3384 (2)	0.36804 (19)	0.0602 (9)	
H30A	0.4974	0.3834	0.3950	0.072*	
H30B	0.5042	0.2980	0.3633	0.072*	
C31	0.3972 (3)	0.3061 (2)	0.4179 (2)	0.0667 (11)	
H31A	0.4439	0.2873	0.4682	0.080*	
H31B	0.3566	0.3482	0.4286	0.080*	
C32	0.3283 (3)	0.2393 (2)	0.3758 (2)	0.0642 (10)	
H32A	0.3693	0.1948	0.3705	0.077*	
H32B	0.2879	0.2221	0.4071	0.077*	
C33	0.2587 (3)	0.26442 (19)	0.2949 (2)	0.0537 (8)	
H33A	0.2132	0.3055	0.3004	0.064*	
H33B	0.2177	0.2197	0.2683	0.064*	
C34	0.3193 (2)	0.29517 (16)	0.24557 (17)	0.0389 (6)	
H34A	0.2727	0.3128	0.1947	0.047*	
H34B	0.3597	0.2524	0.2360	0.047*	
C35	0.6341 (3)	0.2047 (2)	0.2061 (3)	0.0632 (10)	
H35	0.6057	0.1574	0.1823	0.076*	
C36	0.6228 (3)	0.2370 (2)	0.2737 (2)	0.0668 (11)	
H36	0.5855	0.2156	0.3028	0.080*	
C37	0.6792 (3)	0.3091 (2)	0.2901 (2)	0.0687 (11)	
H37	0.6856	0.3431	0.3322	0.082*	
C38	0.7228 (3)	0.3197 (2)	0.2324 (3)	0.0691 (11)	
H38	0.7633	0.3622	0.2292	0.083*	
C39	0.6959 (3)	0.2555 (2)	0.1797 (3)	0.0635 (10)	
H39	0.7152	0.2478	0.1356	0.076*	

### Atomic displacement parameters (Å^2^)

**Table:**

	*U*^11^	*U*^22^	*U*^33^	*U*^12^	*U*^13^	*U*^23^
Ru1	0.03591 (14)	0.03186 (13)	0.04509 (15)	0.00501 (9)	0.01884 (10)	0.00734 (9)
Co1	0.0434 (2)	0.02687 (19)	0.0391 (2)	0.00810 (15)	0.01801 (17)	0.00400 (15)
Cl1	0.0557 (5)	0.0404 (4)	0.0578 (5)	0.0007 (3)	0.0344 (4)	0.0092 (3)
P1	0.0381 (4)	0.0284 (3)	0.0365 (4)	−0.0033 (3)	0.0171 (3)	0.0012 (3)
P2	0.0430 (4)	0.0250 (3)	0.0385 (4)	0.0069 (3)	0.0227 (3)	0.0045 (3)
P3	0.0459 (5)	0.0635 (6)	0.0611 (6)	0.0046 (4)	0.0155 (4)	0.0177 (5)
F1	0.0789 (16)	0.0942 (18)	0.0911 (17)	−0.0274 (13)	0.0240 (14)	−0.0058 (14)
F2	0.092 (2)	0.179 (3)	0.094 (2)	−0.032 (2)	−0.0087 (17)	−0.006 (2)
F3	0.127 (3)	0.095 (3)	0.082 (2)	−0.043 (2)	0.0239 (19)	0.0314 (17)
F4	0.117 (3)	0.124 (3)	0.292 (7)	0.051 (3)	0.097 (4)	−0.012 (4)
F5	0.150 (4)	0.190 (5)	0.166 (4)	−0.024 (4)	0.026 (3)	0.126 (4)
F6	0.111 (3)	0.191 (5)	0.128 (4)	0.029 (3)	0.063 (3)	−0.047 (3)
C1	0.0474 (17)	0.0326 (14)	0.0459 (17)	0.0037 (12)	0.0176 (14)	−0.0023 (12)
C2	0.0497 (19)	0.0461 (17)	0.0472 (18)	0.0119 (14)	0.0029 (15)	−0.0088 (14)
C3	0.079 (2)	0.0419 (17)	0.0366 (17)	0.0149 (16)	0.0174 (16)	0.0053 (13)
C4	0.062 (2)	0.0330 (14)	0.0426 (17)	0.0089 (13)	0.0272 (15)	0.0039 (12)
C5	0.0457 (16)	0.0281 (13)	0.0347 (14)	0.0060 (11)	0.0167 (12)	0.0006 (11)
C6	0.0470 (17)	0.0377 (15)	0.0459 (17)	0.0031 (13)	0.0247 (14)	−0.0019 (13)
C7	0.0519 (19)	0.0376 (15)	0.0567 (19)	0.0181 (14)	0.0216 (16)	−0.0027 (14)
C8	0.060 (2)	0.0259 (14)	0.0559 (19)	0.0090 (13)	0.0237 (16)	0.0048 (13)
C9	0.0514 (18)	0.0282 (13)	0.0493 (17)	−0.0018 (12)	0.0259 (15)	0.0002 (12)
C10	0.0444 (16)	0.0262 (12)	0.0386 (15)	0.0014 (11)	0.0201 (13)	−0.0026 (11)
C11	0.0468 (17)	0.0281 (13)	0.0395 (15)	0.0020 (12)	0.0226 (13)	0.0008 (11)
C12	0.0573 (19)	0.0320 (14)	0.0517 (18)	0.0042 (13)	0.0280 (15)	0.0091 (13)
C13	0.074 (2)	0.0442 (18)	0.073 (2)	0.0086 (17)	0.043 (2)	0.0217 (17)
C14	0.074 (2)	0.0316 (16)	0.097 (3)	−0.0104 (16)	0.049 (2)	−0.0031 (17)
C15	0.060 (2)	0.0438 (18)	0.077 (2)	−0.0094 (16)	0.0289 (19)	−0.0124 (17)
C16	0.061 (2)	0.0358 (15)	0.0510 (18)	−0.0004 (14)	0.0235 (16)	−0.0063 (14)
C17	0.071 (2)	0.0437 (17)	0.0494 (19)	0.0183 (16)	0.0405 (17)	0.0063 (15)
C18	0.098 (3)	0.0478 (19)	0.071 (2)	0.0283 (19)	0.059 (2)	0.0128 (17)
C19	0.127 (4)	0.051 (2)	0.113 (4)	0.020 (2)	0.081 (3)	−0.005 (2)
C20	0.111 (4)	0.070 (2)	0.079 (3)	0.018 (2)	0.068 (3)	−0.009 (2)
C21	0.117 (4)	0.073 (2)	0.064 (2)	0.024 (2)	0.063 (3)	0.011 (2)
C22	0.080 (3)	0.055 (2)	0.072 (2)	0.0091 (18)	0.050 (2)	0.0057 (18)
C23	0.0467 (18)	0.0396 (16)	0.059 (2)	−0.0109 (14)	0.0180 (16)	−0.0092 (15)
C24	0.076 (3)	0.055 (2)	0.107 (3)	−0.0203 (19)	0.052 (3)	−0.017 (2)
C25	0.066 (3)	0.061 (2)	0.119 (4)	−0.035 (2)	0.037 (3)	−0.025 (2)
C26	0.088 (3)	0.063 (2)	0.125 (4)	−0.035 (2)	0.049 (3)	−0.040 (3)
C27	0.093 (3)	0.064 (2)	0.107 (4)	−0.027 (2)	0.048 (3)	−0.041 (2)
C28	0.061 (2)	0.0498 (19)	0.068 (2)	−0.0166 (16)	0.0323 (18)	−0.0213 (17)
C29	0.0514 (17)	0.0310 (14)	0.0347 (15)	−0.0052 (12)	0.0218 (13)	−0.0013 (11)
C30	0.074 (2)	0.064 (2)	0.0385 (18)	−0.0202 (18)	0.0139 (17)	0.0041 (16)
C31	0.102 (3)	0.060 (2)	0.043 (2)	−0.009 (2)	0.032 (2)	0.0099 (16)
C32	0.093 (3)	0.0506 (19)	0.061 (2)	−0.0102 (19)	0.041 (2)	0.0104 (17)
C33	0.064 (2)	0.0480 (18)	0.061 (2)	−0.0110 (15)	0.0362 (18)	0.0027 (15)
C34	0.0469 (17)	0.0355 (14)	0.0409 (16)	−0.0045 (12)	0.0236 (14)	−0.0028 (12)
C35	0.053 (2)	0.0452 (18)	0.090 (3)	0.0214 (16)	0.024 (2)	0.0171 (19)
C36	0.056 (2)	0.071 (2)	0.071 (3)	0.0219 (19)	0.0193 (19)	0.032 (2)
C37	0.053 (2)	0.079 (3)	0.060 (2)	0.0167 (19)	0.0004 (19)	0.008 (2)
C38	0.0350 (18)	0.073 (3)	0.093 (3)	0.0050 (16)	0.0141 (19)	0.020 (2)
C39	0.050 (2)	0.063 (2)	0.083 (3)	0.0228 (17)	0.0287 (19)	0.016 (2)

### Geometric parameters (Å, °)

**Table:**

Ru1—C35	2.186 (3)	C15—H15A	0.9700
Ru1—C36	2.195 (3)	C15—H15B	0.9700
Ru1—C39	2.199 (3)	C16—H16A	0.9700
Ru1—C38	2.206 (3)	C16—H16B	0.9700
Ru1—C37	2.208 (4)	C17—C22	1.509 (5)
Ru1—P2	2.3282 (8)	C17—C18	1.531 (4)
Ru1—P1	2.3297 (7)	C17—H17	0.97
Ru1—Cl1	2.4372 (7)	C18—C19	1.531 (5)
Co1—C4	2.011 (3)	C18—H18A	0.9700
Co1—C9	2.014 (3)	C18—H18B	0.9700
Co1—C2	2.021 (3)	C19—C20	1.490 (6)
Co1—C3	2.022 (3)	C19—H19A	0.9700
Co1—C1	2.023 (3)	C19—H19B	0.9700
Co1—C10	2.023 (3)	C20—C21	1.508 (5)
Co1—C8	2.028 (3)	C20—H20A	0.9700
Co1—C6	2.033 (3)	C20—H20B	0.9700
Co1—C7	2.036 (3)	C21—C22	1.532 (5)
Co1—C5	2.055 (2)	C21—H21A	0.9700
P1—C10	1.836 (3)	C21—H21B	0.9700
P1—C23	1.864 (3)	C22—H22A	0.9700
P1—C29	1.866 (3)	C22—H22B	0.9700
P2—C5	1.851 (3)	C23—C24	1.484 (5)
P2—C17	1.872 (3)	C23—C28	1.507 (5)
P2—C11	1.880 (3)	C23—H23	0.97
P3—F4'	1.39 (2)	C24—C25	1.543 (5)
P3—F3'	1.47 (2)	C24—H24A	0.9700
P3—F5'	1.48 (3)	C24—H24B	0.9700
P3—F3	1.541 (3)	C25—C26	1.525 (6)
P3—F4	1.541 (4)	C25—H25A	0.9700
P3—F6'	1.55 (3)	C25—H25B	0.9700
P3—F6	1.564 (4)	C26—C27	1.456 (6)
P3—F5	1.568 (4)	C26—H26A	0.9700
P3—F2	1.576 (3)	C26—H26B	0.9700
P3—F1	1.586 (3)	C27—C28	1.503 (5)
C1—C2	1.421 (4)	C27—H27A	0.9700
C1—C5	1.437 (4)	C27—H27B	0.9700
C1—H1	0.9300	C28—H28A	0.9700
C2—C3	1.413 (5)	C28—H28B	0.9700
C2—H2	0.9300	C29—C30	1.520 (4)
C3—C4	1.405 (4)	C29—C34	1.532 (4)
C3—H3	0.9300	C29—H29	0.97
C4—C5	1.433 (4)	C30—C31	1.529 (5)
C4—H4	0.9300	C30—H30A	0.9700
C6—C7	1.417 (4)	C30—H30B	0.9700
C6—C10	1.426 (4)	C31—C32	1.510 (5)
C6—H6	0.9300	C31—H31A	0.9700
C7—C8	1.401 (4)	C31—H31B	0.9700
C7—H7	0.9300	C32—C33	1.507 (5)
C8—C9	1.420 (4)	C32—H32A	0.9700
C8—H8	0.9300	C32—H32B	0.9700
C9—C10	1.428 (4)	C33—C34	1.515 (4)
C9—H9	0.9300	C33—H33A	0.9700
C11—C16	1.528 (4)	C33—H33B	0.9700
C11—C12	1.541 (4)	C34—H34A	0.9700
C11—H11	0.97	C34—H34B	0.9700
C12—C13	1.525 (4)	C35—C36	1.390 (5)
C12—H12A	0.9700	C35—C39	1.413 (5)
C12—H12B	0.9700	C35—H35	0.9300
C13—C14	1.509 (5)	C36—C37	1.428 (5)
C13—H13A	0.9700	C36—H36	0.9300
C13—H13B	0.9700	C37—C38	1.385 (6)
C14—C15	1.519 (5)	C37—H37	0.9300
C14—H14A	0.9700	C38—C39	1.405 (5)
C14—H14B	0.9700	C38—H38	0.9300
C15—C16	1.529 (4)	C39—H39	0.9300
			
C35—Ru1—C36	37.00 (14)	C10—C9—H9	125.8
C35—Ru1—C39	37.60 (13)	Co1—C9—H9	126.2
C36—Ru1—C39	62.51 (15)	C6—C10—C9	106.7 (2)
C35—Ru1—C38	62.18 (15)	C6—C10—P1	128.5 (2)
C36—Ru1—C38	62.32 (15)	C9—C10—P1	124.7 (2)
C39—Ru1—C38	37.20 (14)	C6—C10—Co1	69.78 (17)
C35—Ru1—C37	62.14 (15)	C9—C10—Co1	68.95 (16)
C36—Ru1—C37	37.85 (14)	P1—C10—Co1	127.72 (13)
C39—Ru1—C37	61.93 (16)	C16—C11—C12	110.4 (2)
C38—Ru1—C37	36.56 (15)	C16—C11—P2	116.2 (2)
C35—Ru1—P2	93.77 (11)	C12—C11—P2	115.0 (2)
C36—Ru1—P2	114.70 (11)	C16—C11—H11	104.6
C39—Ru1—P2	107.88 (11)	C12—C11—H11	104.6
C38—Ru1—P2	144.42 (12)	P2—C11—H11	104.6
C37—Ru1—P2	152.44 (11)	C13—C12—C11	110.9 (3)
C35—Ru1—P1	137.89 (11)	C13—C12—H12A	109.4
C36—Ru1—P1	102.56 (11)	C11—C12—H12A	109.4
C39—Ru1—P1	153.98 (12)	C13—C12—H12B	109.4
C38—Ru1—P1	117.82 (12)	C11—C12—H12B	109.4
C37—Ru1—P1	92.97 (12)	H12A—C12—H12B	108.0
P2—Ru1—P1	97.67 (3)	C14—C13—C12	111.8 (3)
C35—Ru1—Cl1	129.99 (11)	C14—C13—H13A	109.2
C36—Ru1—Cl1	150.32 (11)	C12—C13—H13A	109.2
C39—Ru1—Cl1	94.46 (10)	C14—C13—H13B	109.2
C38—Ru1—Cl1	88.00 (11)	C12—C13—H13B	109.2
C37—Ru1—Cl1	116.14 (12)	H13A—C13—H13B	107.9
P2—Ru1—Cl1	89.17 (3)	C13—C14—C15	111.6 (3)
P1—Ru1—Cl1	90.70 (3)	C13—C14—H14A	109.3
C4—Co1—C9	105.38 (13)	C15—C14—H14A	109.3
C4—Co1—C2	69.05 (14)	C13—C14—H14B	109.3
C9—Co1—C2	158.76 (13)	C15—C14—H14B	109.3
C4—Co1—C3	40.76 (13)	H14A—C14—H14B	108.0
C9—Co1—C3	121.73 (13)	C14—C15—C16	110.9 (3)
C2—Co1—C3	40.91 (14)	C14—C15—H15A	109.5
C4—Co1—C1	69.05 (12)	C16—C15—H15A	109.5
C9—Co1—C1	157.84 (12)	C14—C15—H15B	109.5
C2—Co1—C1	41.14 (12)	C16—C15—H15B	109.5
C3—Co1—C1	68.85 (13)	H15A—C15—H15B	108.1
C4—Co1—C10	124.76 (12)	C11—C16—C15	110.7 (3)
C9—Co1—C10	41.43 (11)	C11—C16—H16A	109.5
C2—Co1—C10	158.10 (13)	C15—C16—H16A	109.5
C3—Co1—C10	160.39 (14)	C11—C16—H16B	109.5
C1—Co1—C10	123.08 (11)	C15—C16—H16B	109.5
C4—Co1—C8	117.80 (12)	H16A—C16—H16B	108.1
C9—Co1—C8	41.14 (12)	C22—C17—C18	107.9 (3)
C2—Co1—C8	121.91 (13)	C22—C17—P2	117.0 (2)
C3—Co1—C8	103.87 (13)	C18—C17—P2	113.3 (2)
C1—Co1—C8	160.71 (12)	C22—C17—H17	106)
C10—Co1—C8	69.52 (12)	C18—C17—H17	106
C4—Co1—C6	163.88 (12)	P2—C17—H17	106
C9—Co1—C6	68.93 (12)	C19—C18—C17	111.5 (3)
C2—Co1—C6	121.71 (14)	C19—C18—H18A	109.3
C3—Co1—C6	155.12 (13)	C17—C18—H18A	109.3
C1—Co1—C6	110.20 (12)	C19—C18—H18B	109.3
C10—Co1—C6	41.18 (11)	C17—C18—H18B	109.3
C8—Co1—C6	68.50 (12)	H18A—C18—H18B	108.0
C4—Co1—C7	152.84 (12)	C20—C19—C18	111.3 (3)
C9—Co1—C7	68.67 (13)	C20—C19—H19A	109.4
C2—Co1—C7	106.45 (13)	C18—C19—H19A	109.4
C3—Co1—C7	118.31 (13)	C20—C19—H19B	109.4
C1—Co1—C7	126.10 (13)	C18—C19—H19B	109.4
C10—Co1—C7	69.24 (12)	H19A—C19—H19B	108.0
C8—Co1—C7	40.34 (12)	C19—C20—C21	111.3 (3)
C6—Co1—C7	40.76 (12)	C19—C20—H20A	109.4
C4—Co1—C5	41.26 (11)	C21—C20—H20A	109.4
C9—Co1—C5	120.52 (12)	C19—C20—H20B	109.4
C2—Co1—C5	69.61 (12)	C21—C20—H20B	109.4
C3—Co1—C5	69.22 (12)	H20A—C20—H20B	108.0
C1—Co1—C5	41.27 (11)	C20—C21—C22	111.8 (3)
C10—Co1—C5	108.48 (11)	C20—C21—H21A	109.2
C8—Co1—C5	154.65 (12)	C22—C21—H21A	109.3
C6—Co1—C5	127.55 (11)	C20—C21—H21B	109.2
C7—Co1—C5	164.39 (12)	C22—C21—H21B	109.2
C10—P1—C23	98.49 (14)	H21A—C21—H21B	107.9
C10—P1—C29	100.92 (13)	C17—C22—C21	111.4 (3)
C23—P1—C29	100.04 (14)	C17—C22—H22A	109.4
C10—P1—Ru1	119.39 (9)	C21—C22—H22A	109.4
C23—P1—Ru1	114.06 (11)	C17—C22—H22B	109.4
C29—P1—Ru1	120.17 (9)	C21—C22—H22B	109.4
C5—P2—C17	98.20 (14)	H22A—C22—H22B	108.0
C5—P2—C11	97.89 (13)	C24—C23—C28	110.3 (3)
C17—P2—C11	101.89 (14)	C24—C23—P1	116.7 (3)
C5—P2—Ru1	118.51 (9)	C28—C23—P1	116.4 (2)
C17—P2—Ru1	116.82 (13)	C24—C23—H23	104
C11—P2—Ru1	119.63 (10)	C28—C23—H23	104
F4'—P3—F3'	88.3 (17)	P1—C23—H23	103.8
F4'—P3—F5'	83.8 (17)	C23—C24—C25	110.0 (3)
F3'—P3—F5'	172.0 (17)	C23—C24—H24A	109.7
F4'—P3—F3	135.6 (14)	C25—C24—H24A	109.7
F3'—P3—F3	48.1 (12)	C23—C24—H24B	109.7
F5'—P3—F3	139.9 (13)	C25—C24—H24B	109.7
F4'—P3—F4	46.0 (13)	H24A—C24—H24B	108.2
F3'—P3—F4	42.5 (11)	C26—C25—C24	110.6 (4)
F5'—P3—F4	129.7 (13)	C26—C25—H25A	109.5
F3—P3—F4	89.7 (3)	C24—C25—H25A	109.5
F4'—P3—F6'	169.2 (16)	C26—C25—H25B	109.5
F3'—P3—F6'	100.8 (17)	C24—C25—H25B	109.5
F5'—P3—F6'	87.2 (16)	H25A—C25—H25B	108.1
F3—P3—F6'	52.8 (12)	C27—C26—C25	113.9 (3)
F4—P3—F6'	142.2 (13)	C27—C26—H26A	108.8
F4'—P3—F6	131.9 (14)	C25—C26—H26A	108.8
F3'—P3—F6	139.4 (13)	C27—C26—H26B	108.8
F5'—P3—F6	48.3 (13)	C25—C26—H26B	108.8
F3—P3—F6	92.4 (3)	H26A—C26—H26B	107.7
F4—P3—F6	177.8 (3)	C26—C27—C28	114.4 (4)
F6'—P3—F6	40.0 (11)	C26—C27—H27A	108.7
F4'—P3—F5	42.7 (13)	C28—C27—H27A	108.7
F3'—P3—F5	129.8 (12)	C26—C27—H27B	108.7
F5'—P3—F5	42.2 (12)	C28—C27—H27B	108.7
F3—P3—F5	177.6 (3)	H27A—C27—H27B	107.6
F4—P3—F5	88.5 (3)	C27—C28—C23	113.5 (3)
F6'—P3—F5	129.2 (12)	C27—C28—H28A	108.9
F6—P3—F5	89.4 (3)	C23—C28—H28A	108.9
F4'—P3—F2	87.1 (10)	C27—C28—H28B	108.9
F3'—P3—F2	96.5 (9)	C23—C28—H28B	108.9
F5'—P3—F2	84.3 (10)	H28A—C28—H28B	107.7
F3—P3—F2	89.44 (18)	C30—C29—C34	109.4 (2)
F4—P3—F2	89.5 (2)	C30—C29—P1	114.0 (2)
F6'—P3—F2	86.1 (10)	C34—C29—P1	111.99 (19)
F6—P3—F2	91.3 (2)	C30—C29—H29	107.0
F5—P3—F2	92.0 (2)	C34—C29—H29	107.0
F4'—P3—F1	94.7 (10)	P1—C29—H29	107.0
F3'—P3—F1	84.0 (9)	C29—C30—C31	112.7 (3)
F5'—P3—F1	95.4 (10)	C29—C30—H30A	109.1
F3—P3—F1	89.60 (15)	C31—C30—H30A	109.1
F4—P3—F1	92.0 (2)	C29—C30—H30B	109.1
F6'—P3—F1	92.1 (10)	C31—C30—H30B	109.1
F6—P3—F1	87.24 (18)	H30A—C30—H30B	107.8
F5—P3—F1	89.0 (2)	C32—C31—C30	111.0 (3)
F2—P3—F1	178.18 (18)	C32—C31—H31A	109.4
C2—C1—C5	109.0 (3)	C30—C31—H31A	109.4
C2—C1—Co1	69.39 (17)	C32—C31—H31B	109.4
C5—C1—Co1	70.56 (16)	C30—C31—H31B	109.4
C2—C1—H1	125.5	H31A—C31—H31B	108.0
C5—C1—H1	125.5	C33—C32—C31	111.3 (3)
Co1—C1—H1	126.1	C33—C32—H32A	109.4
C3—C2—C1	107.6 (3)	C31—C32—H32A	109.4
C3—C2—Co1	69.58 (19)	C33—C32—H32B	109.4
C1—C2—Co1	69.47 (17)	C31—C32—H32B	109.4
C3—C2—H2	126.2	H32A—C32—H32B	108.0
C1—C2—H2	126.2	C32—C33—C34	110.8 (3)
Co1—C2—H2	126.3	C32—C33—H33A	109.5
C4—C3—C2	108.4 (3)	C34—C33—H33A	109.5
C4—C3—Co1	69.17 (17)	C32—C33—H33B	109.5
C2—C3—Co1	69.51 (18)	C34—C33—H33B	109.5
C4—C3—H3	125.8	H33A—C33—H33B	108.1
C2—C3—H3	125.8	C33—C34—C29	112.5 (2)
Co1—C3—H3	127.1	C33—C34—H34A	109.1
C3—C4—C5	109.4 (3)	C29—C34—H34A	109.1
C3—C4—Co1	70.07 (18)	C33—C34—H34B	109.1
C5—C4—Co1	71.01 (16)	C29—C34—H34B	109.1
C3—C4—H4	125.3	H34A—C34—H34B	107.8
C5—C4—H4	125.3	C36—C35—C39	108.8 (4)
Co1—C4—H4	125.2	C36—C35—Ru1	71.84 (19)
C4—C5—C1	105.6 (2)	C39—C35—Ru1	71.69 (18)
C4—C5—P2	126.2 (2)	C36—C35—H35	125.6
C1—C5—P2	128.2 (2)	C39—C35—H35	125.6
C4—C5—Co1	67.73 (15)	Ru1—C35—H35	122.5
C1—C5—Co1	68.17 (15)	C35—C36—C37	107.1 (4)
P2—C5—Co1	129.80 (15)	C35—C36—Ru1	71.2 (2)
C7—C6—C10	108.4 (3)	C37—C36—Ru1	71.6 (2)
C7—C6—Co1	69.76 (17)	C35—C36—H36	126.4
C10—C6—Co1	69.04 (16)	C37—C36—H36	126.4
C7—C6—H6	125.8	Ru1—C36—H36	122.6
C10—C6—H6	125.8	C38—C37—C36	108.1 (4)
Co1—C6—H6	127.0	C38—C37—Ru1	71.7 (2)
C8—C7—C6	108.4 (3)	C36—C37—Ru1	70.6 (2)
C8—C7—Co1	69.51 (16)	C38—C37—H37	125.9
C6—C7—Co1	69.48 (16)	C36—C37—H37	125.9
C8—C7—H7	125.8	Ru1—C37—H37	123.4
C6—C7—H7	125.8	C37—C38—C39	108.7 (4)
Co1—C7—H7	126.8	C37—C38—Ru1	71.8 (2)
C7—C8—C9	108.1 (3)	C39—C38—Ru1	71.1 (2)
C7—C8—Co1	70.16 (16)	C37—C38—H38	125.6
C9—C8—Co1	68.91 (15)	C39—C38—H38	125.6
C7—C8—H8	125.9	Ru1—C38—H38	123.1
C9—C8—H8	125.9	C38—C39—C35	107.2 (4)
Co1—C8—H8	126.6	C38—C39—Ru1	71.68 (19)
C8—C9—C10	108.3 (3)	C35—C39—Ru1	70.71 (19)
C8—C9—Co1	69.96 (17)	C38—C39—H39	126.4
C10—C9—Co1	69.61 (16)	C35—C39—H39	126.4
C8—C9—H9	125.8	Ru1—C39—H39	122.9
			
C35—Ru1—P1—C10	−150.51 (19)	C5—Co1—C8—C7	−171.8 (3)
C36—Ru1—P1—C10	−163.67 (15)	C4—Co1—C8—C9	−81.5 (2)
C39—Ru1—P1—C10	144.8 (2)	C2—Co1—C8—C9	−162.98 (18)
C38—Ru1—P1—C10	131.21 (16)	C3—Co1—C8—C9	−122.74 (19)
C37—Ru1—P1—C10	159.32 (15)	C1—Co1—C8—C9	172.1 (3)
P2—Ru1—P1—C10	−46.16 (10)	C10—Co1—C8—C9	37.92 (17)
Cl1—Ru1—P1—C10	43.10 (10)	C6—Co1—C8—C9	82.16 (19)
C35—Ru1—P1—C23	93.6 (2)	C7—Co1—C8—C9	119.6 (3)
C36—Ru1—P1—C23	80.41 (17)	C5—Co1—C8—C9	−52.2 (4)
C39—Ru1—P1—C23	28.9 (3)	C7—C8—C9—C10	0.2 (3)
C38—Ru1—P1—C23	15.29 (17)	Co1—C8—C9—C10	−59.22 (19)
C37—Ru1—P1—C23	43.40 (17)	C7—C8—C9—Co1	59.4 (2)
P2—Ru1—P1—C23	−162.08 (13)	C4—Co1—C9—C8	114.87 (18)
Cl1—Ru1—P1—C23	−72.82 (13)	C2—Co1—C9—C8	43.3 (4)
C35—Ru1—P1—C29	−25.1 (2)	C3—Co1—C9—C8	73.8 (2)
C36—Ru1—P1—C29	−38.30 (16)	C1—Co1—C9—C8	−173.1 (3)
C39—Ru1—P1—C29	−89.9 (3)	C10—Co1—C9—C8	−119.5 (2)
C38—Ru1—P1—C29	−103.42 (16)	C6—Co1—C9—C8	−81.03 (19)
C37—Ru1—P1—C29	−75.31 (16)	C7—Co1—C9—C8	−37.16 (18)
P2—Ru1—P1—C29	79.21 (11)	C5—Co1—C9—C8	156.89 (18)
Cl1—Ru1—P1—C29	168.47 (11)	C4—Co1—C9—C10	−125.59 (17)
C35—Ru1—P2—C5	179.85 (15)	C2—Co1—C9—C10	162.8 (3)
C36—Ru1—P2—C5	148.14 (16)	C3—Co1—C9—C10	−166.69 (17)
C39—Ru1—P2—C5	−144.54 (15)	C1—Co1—C9—C10	−53.5 (4)
C38—Ru1—P2—C5	−135.5 (2)	C8—Co1—C9—C10	119.5 (2)
C37—Ru1—P2—C5	152.3 (3)	C6—Co1—C9—C10	38.51 (16)
P1—Ru1—P2—C5	40.48 (11)	C7—Co1—C9—C10	82.38 (18)
Cl1—Ru1—P2—C5	−50.12 (11)	C5—Co1—C9—C10	−83.57 (18)
C35—Ru1—P2—C17	−62.99 (15)	C7—C6—C10—C9	0.5 (3)
C36—Ru1—P2—C17	−94.71 (16)	Co1—C6—C10—C9	59.32 (19)
C39—Ru1—P2—C17	−27.38 (15)	C7—C6—C10—P1	178.5 (2)
C38—Ru1—P2—C17	−18.4 (2)	Co1—C6—C10—P1	−122.7 (2)
C37—Ru1—P2—C17	−90.6 (3)	C7—C6—C10—Co1	−58.8 (2)
P1—Ru1—P2—C17	157.63 (11)	C8—C9—C10—C6	−0.4 (3)
Cl1—Ru1—P2—C17	67.04 (11)	Co1—C9—C10—C6	−59.86 (19)
C35—Ru1—P2—C11	60.60 (15)	C8—C9—C10—P1	−178.5 (2)
C36—Ru1—P2—C11	28.88 (16)	Co1—C9—C10—P1	122.1 (2)
C39—Ru1—P2—C11	96.21 (15)	C8—C9—C10—Co1	59.4 (2)
C38—Ru1—P2—C11	105.2 (2)	C23—P1—C10—C6	−110.6 (3)
C37—Ru1—P2—C11	33.0 (3)	C29—P1—C10—C6	−8.6 (3)
P1—Ru1—P2—C11	−78.78 (10)	Ru1—P1—C10—C6	125.5 (2)
Cl1—Ru1—P2—C11	−169.37 (10)	C23—P1—C10—C9	67.0 (3)
C4—Co1—C1—C2	−81.7 (2)	C29—P1—C10—C9	169.1 (2)
C9—Co1—C1—C2	−160.9 (3)	Ru1—P1—C10—C9	−56.8 (3)
C3—Co1—C1—C2	−37.9 (2)	C23—P1—C10—Co1	156.09 (19)
C10—Co1—C1—C2	159.63 (19)	C29—P1—C10—Co1	−101.89 (18)
C8—Co1—C1—C2	33.0 (5)	Ru1—P1—C10—Co1	32.2 (2)
C6—Co1—C1—C2	115.5 (2)	C4—Co1—C10—C6	−169.32 (16)
C7—Co1—C1—C2	72.4 (2)	C9—Co1—C10—C6	118.0 (2)
C5—Co1—C1—C2	−120.1 (3)	C2—Co1—C10—C6	−45.3 (4)
C4—Co1—C1—C5	38.37 (17)	C3—Co1—C10—C6	153.7 (3)
C9—Co1—C1—C5	−40.8 (4)	C1—Co1—C10—C6	−83.18 (19)
C2—Co1—C1—C5	120.1 (3)	C8—Co1—C10—C6	80.39 (17)
C3—Co1—C1—C5	82.19 (18)	C7—Co1—C10—C6	37.16 (17)
C10—Co1—C1—C5	−80.27 (19)	C5—Co1—C10—C6	−126.45 (16)
C8—Co1—C1—C5	153.1 (4)	C4—Co1—C10—C9	72.63 (19)
C6—Co1—C1—C5	−124.42 (17)	C2—Co1—C10—C9	−163.3 (3)
C7—Co1—C1—C5	−167.49 (17)	C3—Co1—C10—C9	35.7 (4)
C5—C1—C2—C3	−0.3 (3)	C1—Co1—C10—C9	158.77 (17)
Co1—C1—C2—C3	59.4 (2)	C8—Co1—C10—C9	−37.66 (17)
C5—C1—C2—Co1	−59.6 (2)	C6—Co1—C10—C9	−118.0 (2)
C4—Co1—C2—C3	−37.19 (17)	C7—Co1—C10—C9	−80.89 (18)
C9—Co1—C2—C3	41.2 (4)	C5—Co1—C10—C9	115.51 (17)
C1—Co1—C2—C3	−118.9 (3)	C4—Co1—C10—P1	−45.6 (2)
C10—Co1—C2—C3	−170.4 (3)	C9—Co1—C10—P1	−118.3 (3)
C8—Co1—C2—C3	73.3 (2)	C2—Co1—C10—P1	78.4 (4)
C6—Co1—C2—C3	156.25 (17)	C3—Co1—C10—P1	−82.6 (4)
C7—Co1—C2—C3	114.49 (19)	C1—Co1—C10—P1	40.5 (2)
C5—Co1—C2—C3	−81.43 (19)	C8—Co1—C10—P1	−155.9 (2)
C4—Co1—C2—C1	81.74 (19)	C6—Co1—C10—P1	123.7 (3)
C9—Co1—C2—C1	160.1 (3)	C7—Co1—C10—P1	160.8 (2)
C3—Co1—C2—C1	118.9 (3)	C5—Co1—C10—P1	−2.8 (2)
C10—Co1—C2—C1	−51.4 (4)	C5—P2—C11—C16	62.5 (2)
C8—Co1—C2—C1	−167.77 (18)	C17—P2—C11—C16	−37.7 (2)
C6—Co1—C2—C1	−84.8 (2)	Ru1—P2—C11—C16	−168.25 (18)
C7—Co1—C2—C1	−126.58 (19)	C5—P2—C11—C12	−166.4 (2)
C5—Co1—C2—C1	37.50 (17)	C17—P2—C11—C12	93.5 (2)
C1—C2—C3—C4	−0.9 (3)	Ru1—P2—C11—C12	−37.1 (2)
Co1—C2—C3—C4	58.4 (2)	C16—C11—C12—C13	−55.5 (3)
C1—C2—C3—Co1	−59.3 (2)	P2—C11—C12—C13	170.6 (2)
C9—Co1—C3—C4	76.1 (2)	C11—C12—C13—C14	54.8 (4)
C2—Co1—C3—C4	−120.2 (3)	C12—C13—C14—C15	−55.2 (4)
C1—Co1—C3—C4	−82.04 (19)	C13—C14—C15—C16	56.0 (4)
C10—Co1—C3—C4	49.1 (4)	C12—C11—C16—C15	56.8 (3)
C8—Co1—C3—C4	116.71 (19)	P2—C11—C16—C15	−169.9 (2)
C6—Co1—C3—C4	−174.7 (3)	C14—C15—C16—C11	−57.0 (3)
C7—Co1—C3—C4	157.35 (18)	C5—P2—C17—C22	74.2 (3)
C5—Co1—C3—C4	−37.70 (18)	C11—P2—C17—C22	174.1 (3)
C4—Co1—C3—C2	120.2 (3)	Ru1—P2—C17—C22	−53.6 (3)
C9—Co1—C3—C2	−163.71 (17)	C5—P2—C17—C18	−159.4 (3)
C1—Co1—C3—C2	38.12 (17)	C11—P2—C17—C18	−59.5 (3)
C10—Co1—C3—C2	169.3 (3)	Ru1—P2—C17—C18	72.8 (3)
C8—Co1—C3—C2	−123.12 (19)	C22—C17—C18—C19	−58.4 (4)
C6—Co1—C3—C2	−54.5 (4)	P2—C17—C18—C19	170.5 (3)
C7—Co1—C3—C2	−82.5 (2)	C17—C18—C19—C20	57.4 (5)
C5—Co1—C3—C2	82.46 (19)	C18—C19—C20—C21	−53.9 (5)
C2—C3—C4—C5	1.8 (3)	C19—C20—C21—C22	53.8 (5)
Co1—C3—C4—C5	60.4 (2)	C18—C17—C22—C21	57.8 (4)
C2—C3—C4—Co1	−58.6 (2)	P2—C17—C22—C21	−173.1 (3)
C9—Co1—C4—C3	−121.1 (2)	C20—C21—C22—C17	−56.8 (5)
C2—Co1—C4—C3	37.32 (19)	C10—P1—C23—C24	−88.4 (3)
C1—Co1—C4—C3	81.5 (2)	C29—P1—C23—C24	168.8 (3)
C10—Co1—C4—C3	−162.01 (18)	Ru1—P1—C23—C24	39.2 (3)
C8—Co1—C4—C3	−78.6 (2)	C10—P1—C23—C28	44.8 (3)
C6—Co1—C4—C3	171.9 (4)	C29—P1—C23—C28	−58.0 (3)
C7—Co1—C4—C3	−48.0 (4)	Ru1—P1—C23—C28	172.4 (2)
C5—Co1—C4—C3	119.9 (3)	C28—C23—C24—C25	59.1 (4)
C9—Co1—C4—C5	119.02 (18)	P1—C23—C24—C25	−165.0 (3)
C2—Co1—C4—C5	−82.57 (19)	C23—C24—C25—C26	−57.1 (5)
C3—Co1—C4—C5	−119.9 (3)	C24—C25—C26—C27	50.3 (6)
C1—Co1—C4—C5	−38.37 (17)	C25—C26—C27—C28	−45.6 (6)
C10—Co1—C4—C5	78.1 (2)	C26—C27—C28—C23	47.5 (5)
C8—Co1—C4—C5	161.46 (18)	C24—C23—C28—C27	−54.3 (5)
C6—Co1—C4—C5	52.0 (5)	P1—C23—C28—C27	169.7 (3)
C7—Co1—C4—C5	−167.8 (3)	C10—P1—C29—C30	−155.5 (2)
C3—C4—C5—C1	−1.9 (3)	C23—P1—C29—C30	−54.7 (3)
Co1—C4—C5—C1	57.90 (18)	Ru1—P1—C29—C30	70.8 (2)
C3—C4—C5—P2	176.3 (2)	C10—P1—C29—C34	79.6 (2)
Co1—C4—C5—P2	−123.9 (2)	C23—P1—C29—C34	−179.7 (2)
C3—C4—C5—Co1	−59.8 (2)	Ru1—P1—C29—C34	−54.1 (2)
C2—C1—C5—C4	1.3 (3)	C34—C29—C30—C31	−53.5 (4)
Co1—C1—C5—C4	−57.61 (18)	P1—C29—C30—C31	−179.8 (2)
C2—C1—C5—P2	−176.9 (2)	C29—C30—C31—C32	54.9 (4)
Co1—C1—C5—P2	124.2 (2)	C30—C31—C32—C33	−55.5 (4)
C2—C1—C5—Co1	58.92 (19)	C31—C32—C33—C34	56.5 (4)
C17—P2—C5—C4	−58.3 (3)	C32—C33—C34—C29	−56.6 (4)
C11—P2—C5—C4	−161.6 (2)	C30—C29—C34—C33	54.5 (4)
Ru1—P2—C5—C4	68.4 (3)	P1—C29—C34—C33	−178.1 (2)
C17—P2—C5—C1	119.5 (3)	C39—Ru1—C35—C36	117.8 (4)
C11—P2—C5—C1	16.2 (3)	C38—Ru1—C35—C36	80.1 (3)
Ru1—P2—C5—C1	−113.8 (2)	C37—Ru1—C35—C36	38.5 (2)
C17—P2—C5—Co1	−148.2 (2)	P2—Ru1—C35—C36	−127.5 (2)
C11—P2—C5—Co1	108.5 (2)	P1—Ru1—C35—C36	−21.7 (3)
Ru1—P2—C5—Co1	−21.6 (2)	Cl1—Ru1—C35—C36	140.5 (2)
C9—Co1—C5—C4	−78.2 (2)	C36—Ru1—C35—C39	−117.8 (4)
C2—Co1—C5—C4	81.1 (2)	C38—Ru1—C35—C39	−37.7 (2)
C3—Co1—C5—C4	37.3 (2)	C37—Ru1—C35—C39	−79.3 (3)
C1—Co1—C5—C4	118.5 (3)	P2—Ru1—C35—C39	114.7 (2)
C10—Co1—C5—C4	−122.05 (19)	P1—Ru1—C35—C39	−139.5 (2)
C8—Co1—C5—C4	−41.1 (4)	Cl1—Ru1—C35—C39	22.7 (3)
C6—Co1—C5—C4	−163.97 (18)	C39—C35—C36—C37	−0.4 (4)
C7—Co1—C5—C4	159.1 (4)	Ru1—C35—C36—C37	−62.9 (2)
C4—Co1—C5—C1	−118.5 (3)	C39—C35—C36—Ru1	62.5 (2)
C9—Co1—C5—C1	163.36 (17)	C39—Ru1—C36—C35	−37.5 (2)
C2—Co1—C5—C1	−37.39 (18)	C38—Ru1—C36—C35	−79.7 (3)
C3—Co1—C5—C1	−81.22 (19)	C37—Ru1—C36—C35	−116.2 (4)
C10—Co1—C5—C1	119.46 (17)	P2—Ru1—C36—C35	60.7 (2)
C8—Co1—C5—C1	−159.5 (3)	P1—Ru1—C36—C35	165.3 (2)
C6—Co1—C5—C1	77.5 (2)	Cl1—Ru1—C36—C35	−80.1 (3)
C7—Co1—C5—C1	40.6 (5)	C35—Ru1—C36—C37	116.2 (4)
C4—Co1—C5—P2	119.3 (3)	C39—Ru1—C36—C37	78.8 (3)
C9—Co1—C5—P2	41.2 (2)	C38—Ru1—C36—C37	36.6 (2)
C2—Co1—C5—P2	−159.6 (2)	P2—Ru1—C36—C37	176.9 (2)
C3—Co1—C5—P2	156.6 (2)	P1—Ru1—C36—C37	−78.5 (2)
C1—Co1—C5—P2	−122.2 (3)	Cl1—Ru1—C36—C37	36.1 (4)
C10—Co1—C5—P2	−2.7 (2)	C35—C36—C37—C38	0.4 (4)
C8—Co1—C5—P2	78.3 (3)	Ru1—C36—C37—C38	−62.2 (2)
C6—Co1—C5—P2	−44.6 (3)	C35—C36—C37—Ru1	62.7 (2)
C7—Co1—C5—P2	−81.6 (5)	C35—Ru1—C37—C38	80.0 (3)
C4—Co1—C6—C7	153.4 (4)	C36—Ru1—C37—C38	117.6 (4)
C9—Co1—C6—C7	81.4 (2)	C39—Ru1—C37—C38	37.2 (2)
C2—Co1—C6—C7	−78.0 (2)	P2—Ru1—C37—C38	111.6 (3)
C3—Co1—C6—C7	−39.2 (4)	P1—Ru1—C37—C38	−135.6 (2)
C1—Co1—C6—C7	−122.32 (19)	Cl1—Ru1—C37—C38	−43.4 (3)
C10—Co1—C6—C7	120.1 (2)	C35—Ru1—C37—C36	−37.6 (2)
C8—Co1—C6—C7	37.07 (19)	C39—Ru1—C37—C36	−80.4 (2)
C5—Co1—C6—C7	−165.66 (19)	C38—Ru1—C37—C36	−117.6 (4)
C4—Co1—C6—C10	33.2 (5)	P2—Ru1—C37—C36	−6.1 (4)
C9—Co1—C6—C10	−38.75 (16)	P1—Ru1—C37—C36	106.7 (2)
C2—Co1—C6—C10	161.84 (16)	Cl1—Ru1—C37—C36	−161.0 (2)
C3—Co1—C6—C10	−159.3 (3)	C36—C37—C38—C39	−0.3 (4)
C1—Co1—C6—C10	117.57 (16)	Ru1—C37—C38—C39	−61.9 (2)
C8—Co1—C6—C10	−83.05 (17)	C36—C37—C38—Ru1	61.5 (2)
C7—Co1—C6—C10	−120.1 (2)	C35—Ru1—C38—C37	−79.9 (3)
C5—Co1—C6—C10	74.2 (2)	C36—Ru1—C38—C37	−37.9 (2)
C10—C6—C7—C8	−0.4 (3)	C39—Ru1—C38—C37	−118.0 (3)
Co1—C6—C7—C8	−58.8 (2)	P2—Ru1—C38—C37	−132.3 (2)
C10—C6—C7—Co1	58.4 (2)	P1—Ru1—C38—C37	52.2 (3)
C4—Co1—C7—C8	−44.2 (4)	Cl1—Ru1—C38—C37	141.9 (2)
C9—Co1—C7—C8	37.88 (18)	C35—Ru1—C38—C39	38.1 (2)
C2—Co1—C7—C8	−120.2 (2)	C36—Ru1—C38—C39	80.2 (2)
C3—Co1—C7—C8	−77.6 (2)	C37—Ru1—C38—C39	118.0 (3)
C1—Co1—C7—C8	−161.08 (19)	P2—Ru1—C38—C39	−14.3 (3)
C10—Co1—C7—C8	82.4 (2)	P1—Ru1—C38—C39	170.20 (19)
C6—Co1—C7—C8	119.9 (3)	Cl1—Ru1—C38—C39	−100.1 (2)
C5—Co1—C7—C8	166.8 (4)	C37—C38—C39—C35	0.1 (4)
C4—Co1—C7—C6	−164.2 (3)	Ru1—C38—C39—C35	−62.2 (2)
C9—Co1—C7—C6	−82.06 (19)	C37—C38—C39—Ru1	62.3 (2)
C2—Co1—C7—C6	119.8 (2)	C36—C35—C39—C38	0.2 (4)
C3—Co1—C7—C6	162.41 (19)	Ru1—C35—C39—C38	62.8 (2)
C1—Co1—C7—C6	79.0 (2)	C36—C35—C39—Ru1	−62.6 (2)
C10—Co1—C7—C6	−37.52 (17)	C35—Ru1—C39—C38	−116.5 (4)
C8—Co1—C7—C6	−119.9 (3)	C36—Ru1—C39—C38	−79.6 (3)
C5—Co1—C7—C6	46.9 (5)	C37—Ru1—C39—C38	−36.6 (2)
C6—C7—C8—C9	0.2 (3)	P2—Ru1—C39—C38	171.3 (2)
Co1—C7—C8—C9	−58.6 (2)	P1—Ru1—C39—C38	−20.1 (4)
C6—C7—C8—Co1	58.8 (2)	Cl1—Ru1—C39—C38	80.8 (2)
C4—Co1—C8—C7	158.90 (19)	C36—Ru1—C39—C35	36.9 (2)
C9—Co1—C8—C7	−119.6 (3)	C38—Ru1—C39—C35	116.5 (4)
C2—Co1—C8—C7	77.4 (2)	C37—Ru1—C39—C35	79.9 (3)
C3—Co1—C8—C7	117.7 (2)	P2—Ru1—C39—C35	−72.2 (2)
C1—Co1—C8—C7	52.5 (5)	P1—Ru1—C39—C35	96.4 (3)
C10—Co1—C8—C7	−81.7 (2)	Cl1—Ru1—C39—C35	−162.8 (2)
C6—Co1—C8—C7	−37.45 (19)		

### Hydrogen-bond geometry (Å, °)

**Table:**

*D*—H···*A*	*D*—H	H···*A*	*D*···*A*	*D*—H···*A*
C9—H9···Cl1	0.93	2.65	3.284 (3)	127
C6—H6···F1^i^	0.93	2.32	3.176 (4)	152
C3—H3···F4^ii^	0.93	2.47	3.181 (6)	134

Symmetry codes: (i) *x*−1, *y*, *z*; (ii) −*x*+1, −*y*+1, −*z*.
